# Laurin-Sandrow syndrome

**DOI:** 10.11604/pamj.2021.40.115.28167

**Published:** 2021-10-22

**Authors:** Pragadeesh Palaniappan, Krishna Prasanth Baalann

**Affiliations:** 1 Department of Community Medicine, Sree Balaji Medical College and Hospital, Biher, Chennai, India

**Keywords:** Mirror hands, mirror feet, nasal anomalies, Laurin-Sandrow syndrome

## Image in medicine

Polydactyly is a typical abnormality, happening both as a segregated deformity or as component of a syndrome. The presence of mirror polydactyly, however, is rare. One such feature is seen in Laurin-Sandrow syndrome (LSS). Laurin-Sandrow syndrome (LSS) is characterized by complete polysyndactyly of hands, mirror feet and nose anomalies (hypoplasia of the nasal alae and short columella), often associated with ulnar or fibular duplication (and sometimes tibial agenesis). This image describes a case of 2-year-old boy, who came to the clinic with complaints of cough and cold for 1 week duration. The Child was alert, active and feeding well. The child was born at term by normal delivery to a healthy parent. Antenatal period was normal. No systematic illness occurred in the neonatal period. On Examination, there was cup shaped hands with syndactyly on the fingers extending upto the tip. Polysyndactyly was present in the feet, with nine digits on the right foot and ten on the left. Conspicuous nose with short columella, depressed nasal bridge, underdeveloped nasal alae. There are not many cases of LSS revealed in the literature. Hence there are no clear insights regarding the etiopathogenesis. Laurin-Sandrow syndrome is supposed to be an autosomal dominant disorder and the gene associated is said to be limb development membrane Protein 1 (LMBR1). The child was treated for cold and referred to an orthopedician and pediatrician for further management with respect to LSS.

**Figure 1:**
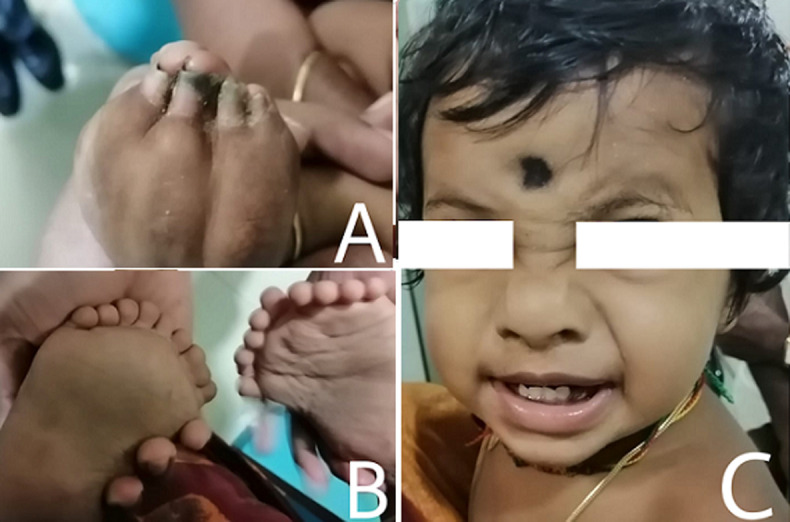
A) cup shaped hands; B) polysyndactyly with nine digits on the foot and ten on the left foot; C) short columella, depressed nasal bridge

